# Prostaglandin treatment is associated with a withdrawal of progesterone and androgen at the receptor level in the uterine cervix

**DOI:** 10.1186/1477-7827-7-116

**Published:** 2009-10-23

**Authors:** Ylva Vladic-Stjernholm, Tomislav Vladic, Chellakkan S Blesson, Gunvor Ekman-Ordeberg, Lena Sahlin

**Affiliations:** 1 Division for Obstetrics and Gynecology, Department of Women's and Children's Health, Karolinska University Hospital and Karolinska Institutet, Stockholm, Sweden; 2 Division for Reproductive Endocrinology, Department of Women's and Children's Health, Karolinska University Hospital and Karolinska Institutet, Stockholm, Sweden

## Abstract

Treatment with prostaglandin(PG)-E_2 _is clinically efficient for cervical priming. The aim of this study was to evaluate the impact of PG-E_2 _on the expression of the progesterone (PR), androgen (AR) and glucocorticoid (GR) receptors in human uterine cervix in prolonged pregnancy.

The study groups were postterm nulliparous women with unripe cervices undergoing cervical priming with PG-E_2 _before labor induction. Responders (n = 12) who delivered vaginally were compared with non-responders (n = 10), who underwent cesarean section due to failure to progress to the active phase of labor. Controls (n = 18) with vaginal partus at a normal gestational age served as a reference group. Cervical levels of PR-A and PR- B isoforms, AR and GR, serum levels of their ligands and sex hormone-binding globulin (SHBG) were quantified.

The responder group displayed lower total PR-AB and AR protein levels as compared to non-responders, and lower PR-B and AR protein levels as compared to controls. In addition, the PR mRNA level was lower in responders as compared to non-responders. The GR protein level did not differ between the groups.

We conclude that successful PG-E_2 _priming was followed by a progesterone and androgen withdrawal at the receptor level in the uterine cervix.

## Background

In clinical practice, cases of maternal or fetal distress necessitate immediate induction of labor. Prostaglandins (PGs) from the E and F series are the main promoters of cervical ripening and myometrial contractility. The influence of PG-E_2 _in promotion of cervical maturation and uterine vasodilatation has been suggested as the primary functions of PGs in human parturition [1]. Local treatment with PG-E_2 _gel is efficient for cervical priming [1,2]. Prolonged pregnancy ≥ 42+0 gestational weeks occurs in 5-12% of pregnancies, predominantly in nulliparous women, exerting increased risks for perinatal mortality and morbidity [3-5]. Prolonged pregnancy is a key indication for cervical priming and induction of labor. The uterine cervix effaces towards the internal os and increases in diameter during the latency phase of labor, and it opens beyond 3-4 cm during the active phase. Late cervical ripening resembles an inflammatory reaction [1,6,7]. Progesterone, testosterone and cortisol are known to have anti-inflammatory properties [1,8,9].

Progesterone withdrawal associated with human parturition is characterized by decreased levels of the total progesterone receptor (PR) and an increased ratio of the inhibitory PR-A isoform to PR-B isoform form in the uterine cervix and myometrium [10-12].

The aim of this study was to evaluate the impact of PG-E_2 _priming on the expression of the PR, androgen (AR) and glucocorticoid (GR) receptors in human uterine cervix in prolonged pregnancy. Serum levels of the receptor ligands, sex hormone-binding globulin (SHBG), and cervical expression of the prostaglandin synthase enzymes constitutive cyclooxygenase (COX)-1 and inducible COX-2 were also determined.

## Methods

### Study patients

Ethics committee approval was obtained before the study (Karolinska University Hospital Ref No. 99-099). All women were healthy, non-smoking, had uncomplicated pregnancies, were without medication and gave informed consent to participate in the study. The study groups were nulliparous women with unripe cervices defined as a Bishop score ≤ 5 points. A Bishop score of ≥ 6 points was the criterion for a ripe cervix according to clinical guidelines [13]. The subjects were treated with PG-E_2 _in viscous gel (Minprostin^® ^Pharmacia, Sweden) for cervical priming and labour induction in postterm pregnancy ≥ 42+0 gestational weeks (Table 1, 2 and 3).

**Table 1:** Clinical data controls.

**Age**	**Gest age**	**Oxytocin**	**Anaesthesia**	**Weight**	**Gender**
27	37+5	+	EDA	3190	M
37	39+6	+	EDA	3965	M
30	39+4	+	EDA	3280	M
34	40+3	+	EDA	3625	M
21	37+0	+	EDA	2595	F
29	40+3	+	EDA	3665	F
20	40+5	+	EDA	3815	F
23	40+6	+	EDA	3065	M
30	39+3	+	EDA	3130	M
32	41+1	+	EDA	3420	M
32	40+5	+	N_2_O	4175	M
28	39+5	+	EDA	3470	M
31	40+1	+	EDA	3590	F
26	40+4	+	EDA	3390	F
31	40+1	+	N_2_O	3540	M
27	39+3	+	EDA	2970	M
26	41+0	+	EDA	2815	F
36	38+6	+	EDA	4055	M
37	40+5	+	EDA	4130	F
					
**30**	**39+6**	**19/19**	**17/19**	**3470**	**F7/M12**

Oxytocin = infusion of oxytocin10 U/glucose 2,5% 500 mL for augmentation of labor. EDA = epidural anaesthesia, N_2_O = inhalation of nitrous oxide 50%/oxygen 50%.

**Table 2:** Clinical data responders.

**Age**	**Gest age**	**BS**	**PG-E**	**Oxytocin**	**Anaesthesia**	**Weight**	**Gender**
21	42+5	2	4	+	EDA	3410	F
31	42+4	2	7	+	EDA	4360	M
39	42+2	3	0,5	+	EDA	2870	F
28	42+3	4	4	+	EDA	3960	F
31	42+2	4	2	+	EDA	3815	F
37	42+5	0	4	+	EDA	3535	F
35	42+5	2	2	+	EDA	4010	M
21	42+4	4	2	+	N_2_O, EDA	3430	M
29	42+3	4	4	+	N_2_O, EDA	3625	M
28	42+1	2	2	+	EDA	4245	M
23	42+4	3	2	+	EDA	3540	M
30	42+1	4	4	+	EDA	3985	M
							
**30**	**42+4**	**3**	**3,1**	**12/12**	**12/12**	**3732**	**F5/M7**

BS = Bishop score (points) before priming, PG-E_2 _= accumulated PG-E_2 _dose (mg), Oxytocin = infusion of oxytocin10 U/glucose 2,5% 500 mL for induction of labor. EDA = epidural anaesthesia, N_2_O = inhalation of nitrous oxide 50%/oxygen 50%.

**Table 3:** Clinical data non-responders.

**Age**	**Gest age**	**BS**	**PG-E**	**Oxytocin**	**Anaesthesia**	**Weight**	**Gender**
32	42+4	2	6	-	EDA	3730	F
24	42+3	2	2	+	EDA	2830	F
31	42+3	2	5	+	SPA	3565	F
29	42+6	0	5,5	-	SPA	5130	M
26	42+5	0	4	-	SPA	3960	F
33	42+4	2	6	+	SPA	2905	M
29	42+4	2	6	+	EDA	3630	F
28	42+5	3	6	+	EDA	4180	M
37	42+4	3	1	+	SPA	5060	M
32	42+6	2	6,5	+	SPA	4930	M
							
**30**	**42+4**	2	**4,7**	**7/10**	**10/10**	**3992**	**F5/M5**

BS = Bishop score (points) before priming, PG-E_2 _= accumulated PG-E_2 _dose (mg), Oxytocin = infusion of oxytocin10 U/glucose 2,5% 500 mL for induction of labor. EDA = epidural anaesthesia, SPA = spinal anaesthesia, N_2_O = inhalation of nitrous oxide 50%/oxygen 50%.

Nulliparous women (n = 18) with spontaneous onset of labor and vaginal partus at a normal gestational length served as a reference control (C) group. They had a median age of 30 years (range 20-37) a median gestational age of 39+6 weeks (range 37+0-41+1). Oxytocin infusion (Syntocinon^® ^10 U/glucose 2,5% 500 mL) for augmentation of labor was administered to all women according to the clinical guidelines [14].

The responders (R) were nulliparous women (n = 12) who delivered vaginally after successful cervical priming with PG-E_2_. They had a median age of 30 years (range 21-39), a median gestational length of 42+4 weeks (range 42+1- 42+5) at partus, and median Bishop score of 3 points (range 0-4) at admission. All 12 women received oxytocin infusion for augmentation of labor.

The non-responders (NR) were nulliparous women (n = 10) who failed to enter the active phase of labor after treatment with PG-E_2 _and therefore delivered by cesarean section [15]. They had a median age of 30 years (range 24-37), a median gestational length of 42+4 weeks (range 42+1- 42+6) at partus, and a median Bishop score of 2 points (range 0-4) before PG-E_2 _treatment. Oxytocin infusion was administered to 7 of 10 women for induction of labor.

It was not possible to obtain a peripheral venous sample from every subject, due to practical reasons or lack of consent. The limited size of the cervical biopsies did not allow for analysis of both mRNA and immunohistochemistry (IHC) in all samples. Thus, the actual number for the mRNA analyses is given in the Results section.

### Sampling procedure

The cervical biopsies were obtained transvaginally from the anterior part of the uterine cervix at the 12 o'clock position, from 10-20 mm depth within 30 min after delivery. The biopsies were divided and one half was immersion-fixed in 4% formaldehyde overnight, stored at 4°C in 70% ethanol and thereafter embedded in paraffin. The other half was immediately frozen at -70°C. Cervical tissue obtained from women after partus at a normal gestational length served as reference material.

### Serum analyses

Peripheral venous samples were drawn immediately after parturition or at cesarean section. They were centrifuged at 3000 × G for 10 min and stored at -20 °C until analyzed.

Serum levels of progesterone and SHBG were determined by direct chemiluminiscence enzyme immunoassay using commercially available kits from Diagnostic Products Corp., Los Angeles, CA (Immulite^®^). Serum testosterone was determined by direct radioimmunoassay using a kit obtained from Diagnostic Products Corp. ("Coat-a-Count^®^"). Serum 5α-DHT was determined after destruction of cross reacting testosterone by oxidative cleavage of the 4-ene double bond with potassium permanganate followed by extraction, using a kit from Diagnostic Systems Laboratories Inc., Webster, Texas, U.S.A. Values obtained by this method were compared with those obtained by a method including extraction with diethyl ether, column chromatography on celite and subsequent radioimmunoassay and an excellent correlation was found [16]. Circulating progesterone binds in < 1%, testosterone in 70%, and its active metabolite 5α-dehydrotestosterone (DHT) in 28%, by high affinity to sex hormone-binding globulin (SHBG) [17]. The free androgen index (FAI) (total T/SHBG × 100) was calculated since the non SHBG-bound fraction is the biologically active fraction of steroid hormones [18].

### RNA preparation and reverse transcription

Total RNA from frozen cervical tissue samples was purified with the RNeasy^® ^kit (Qiagen GmbH, Hilden, Germany) according to a procedure for RNA isolation from fibrous tissues, including a DNase step, as recommended by the manufacturer. Two μg of total RNA from each sample was reverse transcribed at 37°C for 60 min in a final volume of 30 μl with a reaction mixture (Qiagen) containing 1 × RT buffer, dNTP mix (0.5 mM each dNTP), 600 ng random primers (Invitrogen, Paisley, UK), 10 units RNase inhibitor (Superase-In, Ambion, Austin, TX), and 4 U of Omniscript™ reverse transcriptase (Qiagen).

### Real time PCR analysis

The oligonucleotide primers for PR-AB, PR-B, AR and Cyclophilin A are presented in Table 4, as well as their predicted sizes. Real time PCR was performed in a DNA Engine Opticon™ 2 System (MJ Research, Waltham, MA). For PCR, the cDNAs corresponding to 40-100 ng (see Table 2) RNA were added to 10 μl of Quantitect™ SYBR^® ^Green PCR mix (Qiagen) containing HotStarTaq DNA polymerase, PCR buffer, dNTP mixture and 0.3 μM of each oligonucleotide primer in a final volume of 20 μl. The reactions were performed in opaque white 0.2 ml low-profile strip tubes sealed with optical flat caps (TLS-0851, TCS-0803, MJ Research). After initial incubation for 15 min at 95°C, the samples were subjected to 40-44 cycles of 10 s at 94°C, 15-20 s at 56°C (see Table 4) and 20 s at 72°C with a final extension step at 72°C for 5 min. All PCR assays were performed twice. The purity of PCR products was confirmed by a melting curve analysis in all experiments (data not shown). All primers were designed to span an intron/exon boundary or to flank an intron. Thus, amplification of contaminating DNA was eliminated. Each PCR assay included a negative control containing a RNA sample without reverse transcription. The primers were based on the sequences of the human genes. The primer pairs (Table 4) were designed with Primer3 software [19].

**Table 4:** Oligonucleotide primers used for real-time PCR.

**Gene**	**Accession No**	**Primers** **F = forward; R = reverse**	**Position**	**cDNA**	**Annealing step**
PRAB	NM_000926.4 [37]	F: tggaagaaatgactgcatcg R: agcatccagtgctctcacaa	bp 2555-2574 bp 2702-2683 product: 148 bp	40 ng	56°C/15 s
PRB	NM_000926.4 [37]	F: gactgagctgaaggcaaagg R: cgaaacttcaggcaaggtgtc	bp 746-765 bp 890-870 product: 145 bp	40 ng	56°C/15 s
AR	NM_000044.2	F: taccagctcaccaagctcct R: gcttcactgggtgtggaaat	bp 3687-3706 bp 3882-3861 product: 195 bp	100 ng	56°C/20 s
Cyclophilin A	NM_021130.3	F: gtggtgtttggcaaagtgaa R: tcgagttgtccacagtcagc	bp 462-481 bp 577-558 product: 116 bp	40 ng	56°C/20 s

### Quantification of mRNA

To standardize the quantification method, cyclophilin A was selected as the housekeeping gene. The PCR amplification rate and the cycle threshold (Ct) values were analyzed using Opticon Monitor 3.0 software (MJ Research). The values of relative expression of genes of interest were normalized against the cyclophilin A product.

### Immunohistochemical analysis

Immunostaining for the determination of PR-AB, PR-A, PR-B, AR, GR, COX-1 and COX-2 utilizing the avidin-biotin peroxidase complex (ABC) procedure was performed [20]. The 5 μm paraffin sections prepared from cervical biopsies were first dewaxed in Bioclear (Bio-Optica, Milan, Italy), rehydrated and washed with phosphate-buffered saline (PBS; pH 7.4). Thereafter the sections were subjected to microwave antigen retrieval in 0.01 M sodium citrate buffer (pH 6.0) for 10 min and then allowed to cool for 20 min. Subsequently, endogenous peroxidase activity was quenched by immersion in 3% hydrogen peroxide (Merck) in methanol for 10 min at room temperature (RT); followed by blocking non-specific binding of the primary antibody by incubation as shown in Table 5, at RT. The sections were then incubated with the primary antibodies (see Table 5). For the negative controls the primary antibody was replaced by mouse IgG (or in the case of GR rabbit IgG, and for COX-1 and -2 goat IgG) at a corresponding concentration to the antibody it replaced.

**Table 5:** Antibodies used in the study.

**Protein**	**Primary ab, company and order number**	**Type and dilution**	**Inc temp + time**	**Blocking**	**Biotinylated 2 nd Ab all diluted 1:200**	**Buffer**	**Inc time in RT**
PRAB	Zymed Lab Inc, 18-0172	Mc mouse anti human 1:150	RT 60 min	1.5% NHS	horse anti-mouse	1.5% NHS in PBS	45 min
PRA	Novocastra Labs Ltd, NCL-PGR312	Mc mouse anti human 1:500	RT 60 min	1.5% NHS	horse anti-mouse	1.5% NHS in PBS	45 min
PRB	Affinity BioReagents Inc, MA1-411	Mc mouse Anti chick 1:100	RT 60 min	1.5% NHS	horse anti-mouse	1.5% NHS in PBS	45 min
AR	DakoCyto-mation Inc, M3562	Mc mouse anti human 1:100	+4°C o/n	2% NHS	horse anti-mouse	2% NHS in PBS	30 min
GR	Affinity Bio Reagents Inc, PA1-511A	Pc rabbit anti human 1:1000	+4°C o/n	2% NGS	goat anti rabbit	2% NGS in PBS	60 min
COX-1	Santa Cruz Biotechnology Inc, sc 1752	Pc goat anti human 1:100	+37°C 60 min	10% NRS	rabbit anti goat	10% NRS in PBS	30 min
COX-2	Santa Cruz Biotechnology Inc, sc 1745	Pc goat anti human 1:100	+37°C 60 min	10% NRS	rabbit anti goat	10% NRS in PBS	30 min

Mc = monoclonal, Pc = polyclonal

RT = room temperature

biotinylated horse anti-mouse (Vector, Cat# BA-2000)

biotinylated goat anti rabbit (Vector, Cat# BA-1000)

biotinylated rabbit anti goat (Vector, Cat# BA-5000)

The secondary biotinylated antibodies were incubated as shown in Table 5, followed by incubation with an avidin-biotin horseradish peroxidase complex (Vectastain Elite, Cat# PK-6100) for 30 min at RT. The site of the bound enzyme was visualized by the application of 3,3'-diaminobenzidine (DAB kit, Vector, CA), a chromogen which produces a brown, insoluble precipitate when incubated with enzyme. The sections were counterstained with haematoxylin and dehydrated before they were mounted with Pertex (Histolab, Gothenburg).

### Image analysis

A Leica microscope and Sony video camera (Park Ridge, NJ, U.S.A.) connected to a computer with an image analysis system (Leica Imaging System Ltd, Cambridge, UK) was used to assess quantitative values from immunohistochemistry. The quantification of immunostaining was performed as described previously [20]. In short, by using colour discrimination software the total area of positively stained nuclei was measured, and expressed as a ratio of the total area of cell nuclei.

### Manual scoring

Two observers blinded to the identity of the slides performed all the assessments. The staining was evaluated semi-quantitatively using a grading system. The staining intensity was graded on a scale of (0) no staining, (1) faint, (2) moderate or (3) strong staining.

### Statistical analyses

Clinical data were calculated with ANOVA/ANCOVA and significances were evaluated with Scheffe's test. Statistical calculation for serum hormone levels, receptor and mRNA levels was performed by ANOVA on ranks (Kruskal-Wallis test) and significances were evaluated by Dunn's test. Values with different letter designations are significantly different at level p < 0.05.

## Results

### Clinical data

The median maternal age and fetal gender did not differ between the groups. The neonatal weights were, as was expected, significantly higher in the postterm NR and R groups as compared to term C group (p < 0.05).

### Serum levels of steroid hormones and SHBG

No differences were observed for progesterone, total testosterone, 5α-DHT levels or FAI between C (n = 16), R (n = 8) and NR (n = 8) groups. Serum progesterone (medians and range) was 299 (175-563) nmol/L in C, 220 (115-394) in R, and 234 (159-423) in NR. Serum testosterone was 3.06 (1.07-7.84) nmol/L in C, 3.42 (1.72-4.47) in R, and 2.93 (2.13-7.81) in NR. Serum 5α-DHT was 1.02 (0.04-2.42) nmol/L in C, 0.96 (0.57-1.18) in R, and 0.77 (0.58-1.22) in NR. Serum SHBG was significantly lower 301 (248-424) nmol/L in NR (n = 7), as compared to C 512 (304-708) and R 504 (236-644), whereas the FAI (T/SHBG × 100) was comparable between the groups 0.556 (0.187-2.56) in C, 0.770 (0.352-1.75) in R, and 0.834 (0.634-2.60) in NR.

### mRNA levels

**Figure 1:**
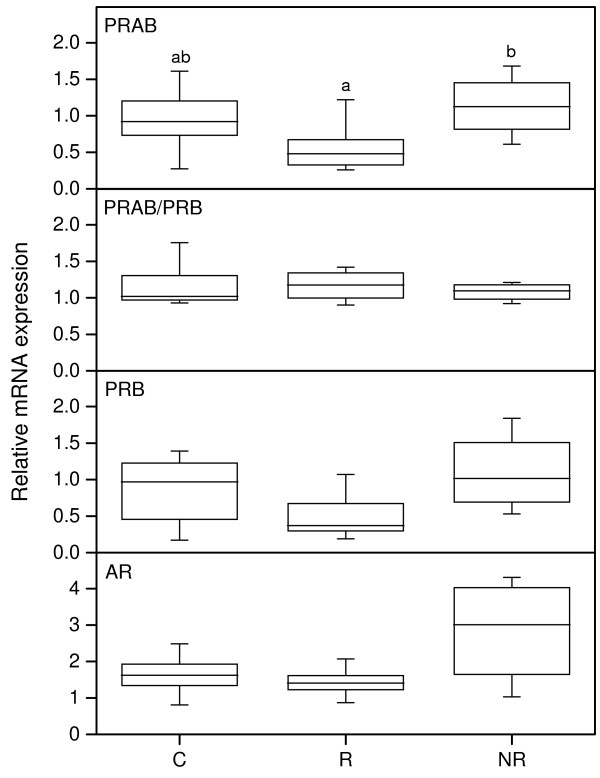
**Real-time PCR results for expression of PR-AB (upper), PR-AB/PR-B (upper middle), PR-B (lower middle) and AR (bottom) mRNAs in human cervix from the C (n = 11), R (n = 10) and NR (n = 4) groups respectively**. The values of relative expression of target genes were normalized against cyclophilin A and displayed in arbitrary units. The ratio of PR-AB/PR-B represents the PR-A expression. The "box-and-whisker plot" represents the median value with 50% of all data falling within the box. The whiskers extend to the 5th and 95th percentiles. Boxes with different letter designations are significantly different, p < 0.05.

The PR-AB mRNA level was lower in responders as compared to the non-responders. The ratio between PR-AB and PR-B represents the PR-A mRNA level which did not change between the groups (Figure 1, upper middle panel). The PRB mRNA showed similar results as the PR-AB mRNA, but differences between groups did not reach significance. The AR mRNA level showed a tendency to be increased in the NR group, but did not reach significance (Figure 1, bottom panel).

### Immunohistochemistry

**Figure 2:**
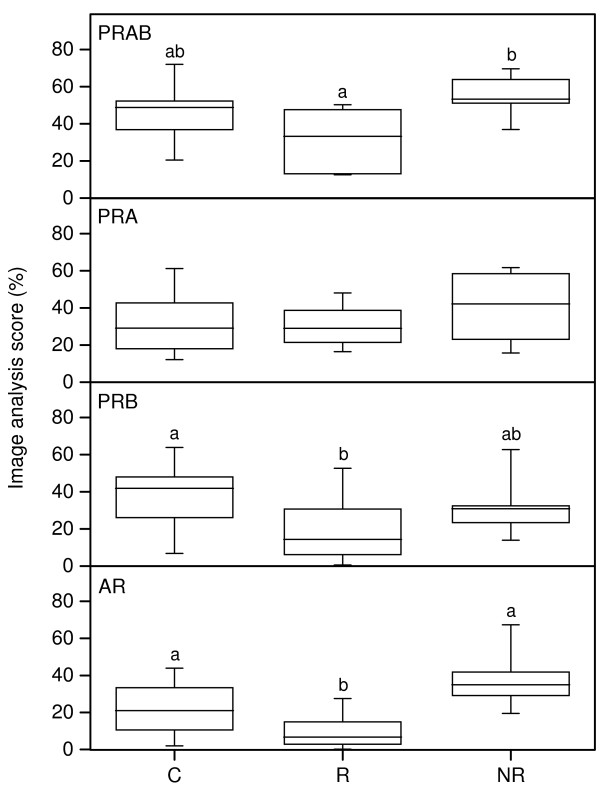
**Immunostaining results for PR-AB (top), PR-A (upper middle), PR-B (lower middle) and AR (bottom) in stroma, as assessed by image analysis in cervical samples from controls (C), responders (R) and non-responders (NR)**. The "box-and-whisker plot" represents the median value with 50% of all data falling within the box. The whiskers extend to the 5th and 95th percentiles. Boxes with different letter designations are significantly different, p < 0.05.

**Figure 3:**
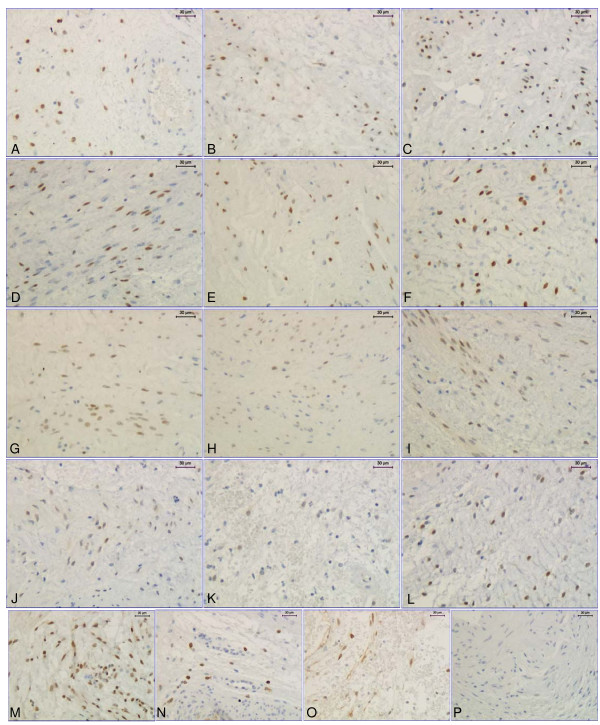
**Representative images of the immunostaining results for PR-AB (A-C), PR-A (D-F), PR-B (G-I), AR (J-L), COX-1 (M), COX-2 (N) and GR (O)**. A negative control is shown for monoclonal antibodies (**P**) where the primary antibody was replaced by an equal amount of mouse IgG. The magnification bars represent 30 μm in all images.

The immunohistochemistry (IHC) scores for nuclear PR-AB (top panel), PR-A (upper middle panel), PR-B (lower middle panel) and AR (bottom panel) protein are presented in Figure 2 and representative images of the IHC results are shown in Figure 3.

The IHC score for PR-AB was lower in responders as compared to non-responders (Figure 2 top panel; Figure 3A-C). The IHC score for PR-B was lower in responders as compared to controls (Figure 2 lower middle panel; Figure 3G-I). The IHC score for PR-A did not differ between study groups.

Immunohistochemical analysis revealed a significantly lower AR expression in the responders as compared to non-responders and controls (Figure 2 bottom panel; Figure 3J-L).

The IHC scores for stromal COX-1, COX-2 and GR did not differ between groups (data not shown). Representative images from immunostaining of COX-1 (M), COX-2 (N) and GR (O) are shown in Figure 3 as well as a negative control (P) where the primary antibody was replaced by an equal amount of mouse IgG.

## Discussion

Prolonged pregnancy constitutes a key indication for cervical priming and induction of labor [3-5]. Local treatment with PG-E_2 _gel has been shown to promote degradation of the extracellular matrix by increasing matrix metalloproteinase (MMP)-1 collagenase activity and by altering the proteoglycan content [2,21,22]. Prostaglandin-E_2 _was reported to induce a functional progesterone withdrawal *in vitro *by increasing the inhibitory PR-A over PR-B expression through the protein kinase (PK)-C pathway in a human myometrial cell line (PHM1-31) [23].

We found that serum levels of the receptor ligands were unchanged between the groups. However, the responder group displayed a lower cervical level of total PR-AB protein as compared to non-responders, and a lower cervical PR-B isoform level as compared to controls. The PR mRNA level was lower in responders as compared to non-responders, whereas no difference was found for the inhibitory PR-A isoform, which acts as a repressor of the PR-B isoform and other steroid receptors such as AR and GR [24]. Thus, decreased PR-AB and PR-B protein levels in responders indicate that PG-E_2 _priming leading to vaginal partus was associated with a nuclear PR withdrawal. The decreased PR-AB level in responders as compared to non-responders could be the consequence of a vaginal delivery. Nevertheless, the decreased PR-B level in responders as compared to controls cannot be explained by a different mode of delivery. Furthermore, PG-E_2 _promotes chemotaxis in neutrophils and macrophages, which enhances leukocyte extravasation into tissues by a synergistic action with chemotactic interleukin (IL)-8 [1,25,26]. This is in accordance with the high leukocyte density and immunostaining for IL-8 observed in responders [27]. Pro-inflammatory cytokines activate the key pro-inflammatory transcription factor nuclear factor (NF)-κB, which was shown to exert a mutual negative interaction with PR [8,28]. The comparable levels of COX-2 between the groups could be explained by the oxytocin treatment, since oxytocin initiates COX-2 gene transcription [29]. In addition, mechanical stretch induces COX-2 activity [30].

Since the cervical AR protein level was lower in responders not only as compared to non-responders who delivered by cesarean sections, but also as compared to controls who delivered spontaneously, the result cannot be explained by a different mode of delivery. The low AR protein level in responders indicates that successful PG-E_2 _priming was followed by an androgen withdrawal at the receptor level. This finding is in accordance with results from *in vitro *studies, in which androgens were reported to attenuate the synthesis of pro-inflammatory cytokines, inhibit MMP-1 production and regulate cervical resistance by altering the proteoglycan content [31-34]. Possible responses to the androgen withdrawal reported here could therefore be events leading to degradation of the extracellular matrix and cervical maturation [21,22,32].

Comparing the results of mRNA and protein for PR-AB, PR-A, PR-B and AR, all showed a similar expression pattern, although only PR-AB displayed significant changes for both mRNA and protein. The lack of significance for mRNA levels of PR-B and AR could be due to the fact that mRNA levels were detected in cervix tissue homogenate whereas the protein levels were determined in stromal nuclei.

The total GR protein level in human uterine cervical stroma and squamous epithelium was decreased after term labor as compared to late pregnancy [35], but no differences were found between the groups in the present study.

In conclusion, successful local PG-E_2 _treatment for cervical priming after prolonged pregnancy was correlated to a progesterone and androgen withdrawal at the receptor level. A comparable progesterone and androgen withdrawal was neither observed in the non-responders, who failed to enter the active phase of labor and underwent cesarean sections, nor in the term control group with spontaneous deliveries. The progesterone withdrawal is in accordance with the previous study [36], whereas the possibility of an androgen withdrawal, to our knowledge, has not been reported previously. We conclude that successful PG-E_2 _priming was followed by a functional progesterone and androgen withdrawal at the receptor level in the uterine cervix. It is possible that treatment with an antiprogestin or an antiandrogen could serve as a supplement to PG-E_2 _priming in non-responders.

## Competing interests

The authors declare that they have no competing interests.

## Authors' contributions

YVS and GEO conceived and designed the study. YVS collected all samples. GEO run the serum samples. TV did the statistics for the clinical data and hormone levels. CSB and LS performed and analysed all mRNA and immunohistochemistry analyses/data, including statistical evaluation. YVS and TV drafted the manuscript. LS and CSB helped to draft the manuscript. All authors read and approved the final manuscript.
